# circMET promotes NSCLC cell proliferation, metastasis, and immune evasion by regulating the miR-145-5p/CXCL3 axis

**DOI:** 10.18632/aging.103392

**Published:** 2020-07-02

**Authors:** Xu Pei, Shi-Wei Chen, Xiang Long, Shu-Qiang Zhu, Bai-Quan Qiu, Kun Lin, Feng Lu, Jian-Jun Xu, Peng-Fei Zhang, Yong-Bing Wu

**Affiliations:** 1 Department of Cardiothoracic Surgery, The Second Affiliated Hospital of Nanchang University, Nanchang, Jiangxi, China; 2 Department of Medical Oncology, Zhongshan Hospital, Fudan University, Shanghai, China

**Keywords:** circMET, NSCLC, miR-145-5p, CXCL3, proliferation

## Abstract

In recent years, circular RNAs (circRNAs) have been increasingly reported to play a crucial role in the proliferation, migration, and invasion of non-small-cell lung cancer (NSCLC) cells. However, the circRNA MET (circMET) oncogenic mechanism that drives NSCLC development and progression remains largely unknown. In this study, the present results demonstrated that circMET expression was significantly higher in NSCLC tissues than in peritumoral tissues using quantitative real-time polymerase chain reaction. Notably, NSCLC patients with a large tumor diameter, poor differentiation and lymphatic metastasis had high RNA levels of circMET. Moreover, high circMET expression served as an independent risk factor for short overall survival (OS) and progression-free survival (PFS) in NSCLC patients. Next, we validated that circMET overexpression can enhance NSCLC cell proliferation, metastasis, and immune evasion in vitro. Mechanistically, our study uncovers that circMET acts as a miR-145-5p sponge to upregulate CXCL3 expression. Collectively, circMET regulates the miR-145-5p/CXCL3 axis and serves as a novel, promising diagnostic and prognostic biomarker in patients with NSCLC.

## INTRODUCTION

Lung cancer is recognized as one of the most common malignant tumors and is the leading cause of cancer-related death worldwide [1]. Based on the statistical data released by the World Health Organization, it is estimated that the number of new cases of NSCLC in 2018 would total 18.1 million, and of these, approximately 1.8 million resulted in death. This toll has been increasing unpredictably worldwide, especially in high-income developed countries [2]. Non-small-cell lung cancer (NSCLC), the main histological type of lung cancer, accounts for approximately 85% of all primary lung cancer cases [3]. Despite progressive advances in NSCLC diagnosis and treatment during the past several decades, it remains a “deadly cancer” and is associated with the highest incidence and mortality rates, with a low 5-year survival rate of only approximately 18% [4, 5]. As is well known, the molecular mechanisms related to the occurrence and progression of NSCLC are complex, multistep pathological processes and have not yet been fully clarified. Therefore, gaining further knowledge of the underlying regulatory mechanisms of NSCLC progression and identifying a predictive biomarker or potential treatment strategy for NSCLC patients is extremely urgent.

Circular RNA (circRNA), a special subtype of noncoding RNA, features a covalently closed continuous loop structure without 5’-3’ polarity or a polyadenylated tail [6, 7]. Unlike traditional linear RNAs, circRNAs have a higher tolerance to exonucleases and tend to be stably and widely expressed in the cytoplasms of various eukaryotic cells [8, 9]. Emerging evidence has indicated that circRNAs can broadly participate in the initiation and progression of various malignant tumors, including NSCLC [10, 11]. Moreover, circRNAs exert their biological effects through diverse mechanisms, such as by acting as miRNA sponges and transcriptional regulators and by interacting with RNA-binding proteins [12]. In our previous study, circFGFR3 was demonstrated to act as a miR-22-3p sponge to regulate galectin-1 expression [13]. Likewise, the oncogenic roles of other circRNAs, such as the circRNAs PTPRA, ARHGAP10, and PTK2, have also frequently been confirmed in NSCLC progression [14–16]. Thus, the aforementioned evidence indicates the potential of circRNAs as novel biomarkers and therapeutic targets. Nonetheless, the underlying molecular mechanisms of circRNA pathway activation are not fully understood on NSCLC progression [17], so the role and contribution of circRNAs remain to be further investigated.

Here, we first analyzed the gene expression profiles of mesenchymal epithelial transition factor receptor (MET)-derived circRNAs in human NSCLC tissues and paired adjacent normal lung tissues. Importantly, we presented the oncogenic role of circMET (also known as hsa_circ_0082003 in circBase), which was significantly upregulated in NSCLC tissues and is closely associated with the poor prognosis of NSCLC patients. Furthermore, we identified that circMET could promote the progression of NSCLC cells by sponging oncogenic miR-145-5p [18, 19] to upregulate chemokine (C-X-C motif) ligand 3 (CXCL3) [20, 21] expression. Therefore, circMET may serve as a novel diagnostic biomarker and promising therapeutic target for NSCLC patients.

## RESULTS

### circMET is a promising diagnostic and prognostic biomarker for NSCLC patients

**Figure 1:**
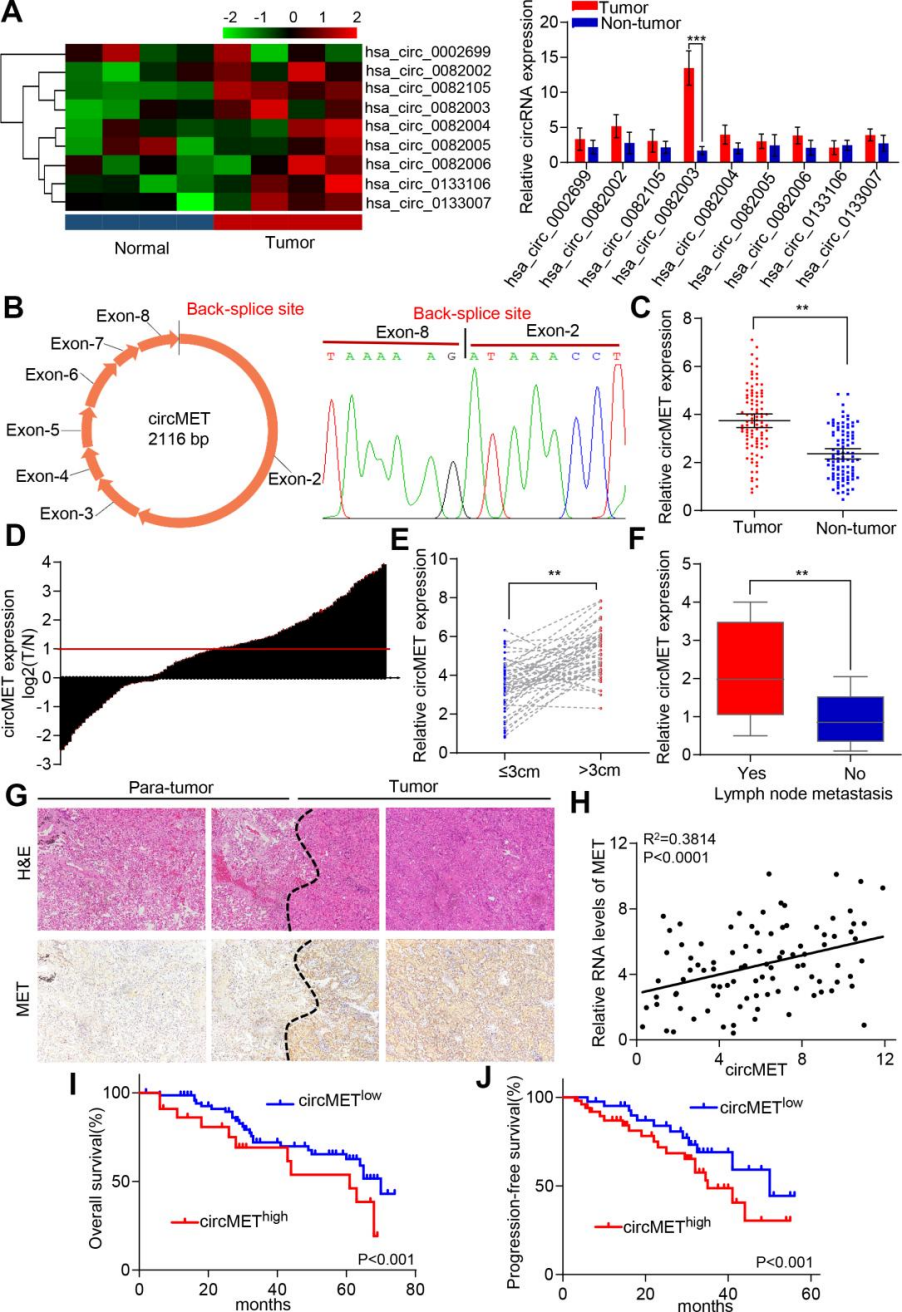
**Identification of circMET in NSCLC tissues and its prognostic significance.** (**A**) Clustered heat map shows the expression profiles of circRNAs derived from the MET gene in four paired NSCLC and adjacent nontumor tissues by qRT-PCR. (**B**) Scheme illustrating the production of circMET. (**C**) and (**D**) circMET was differentially expressed in 94 paired NSCLC tissues and adjacent normal tissues using real-time PCR analysis. (**E**) and (**F**) In total, 94 NSCLC patients were individually divided into low- and high-expression groups according to the clinicopathological characteristics, including tumor size or lymph node metastasis. (**G**) Representative images of serial sections stained with H&E and IHC for MET expression in NSCLC tissues. (**H**) A positive correlation between circMET and MET was observed in NSCLC tissues. (**I**) and (**J**) Prognostic analysis of circMET expression in 94 NSCLC patients. GAPDH was used as an internal control. *P < 0.05, **P < 0.01, *** p < 0.001.

Dysregulation of MET oncogene-mediated cell motility, invasion, and metastasis has been widely documented among many different types of cancer, including NSCLC [22, 23]. Based on this point, we hypothesized that the accumulation of circRNA-derived MET might act as a tumor promoter in NSCLC cells. Therefore, we used circular RNA sequencing data from circBase to identify and screen 9 candidate circRNAs derived from MET genes. To further examine the levels of circRNA expression in NSCLC patients, 4 pairs of NSCLC tissue samples and their corresponding peritumoral tissues were analyzed by qRT-PCR. Among the 9 differentially expressed circRNAs, circMET (hsa_circ_0082003) expression was found to be significantly upregulated in NSCLC tissues relative to that in paired adjacent normal lung tissues (Figure 1A). In addition, the sequence of circMET consisting of 8 exons had a length of 2116 nucleotides (Figure 1B). To investigate the clinical role of circMET in NSCLC patients in detail, we detected the expression of circMET in 94 pairs of NSCLC tissues and matched adjacent nontumor lung tissues. The results indicated that circMET expression was significantly increased in 94 lung cancer tissues (P < 0.001, Figure 1C and 1D). We next sought to investigate the relationship between the circMET expression level in NSCLC tissues and the specific clinicopathological characteristics of NSCLC patients. As shown in Table 1, increased circMET expression in NSCLC was significantly correlated with differentiation, tumor size and lymph node metastasis (Figure 1E and 1F; Table 1). In addition, IHC staining showed that MET expression was significantly higher in NSCLC tumor tissues than in peritumoral tissues, as expected (Figure 1G). Notably, a positive correlation between circMET and MET was observed in the NSCLC tissues (Figure 1H). Moreover, Kaplan-Meier survival curves revealed that patients with circMET^high^ expression were related to poor overall survival (OS) and progression-free survival (PFS) after surgery (Figure 1I and J). Together, these findings suggest that high circMET expression is likely a key promoter in the progression of NSCLC cells and serves as an independent prognostic biomarker for NSCLC patients.

**Table 1:** Correlations between circMET with clinicopathologic features in 94 NSCLCs.

**Variable**	**No. of patients**		P value*
	**circMET^low^**	**circMET^high^**	
Age, year			
≥60	32	27	0.246
<60	20	15	
Gender			
Male	29	22	0.367
Female	23	20	
Smoking			
Yes	38	25	0.523
No	14	17	
Histologic type			
Squamous cell carcinoma	26	24	0.218
Adenocarcinomas	19	16	
Others	7	2	
Tumor diameter(cm)			
≤3	29	11	0.006
>3	23	31	
TNM stage			
I-II	34	14	0.249
III-IV	18	28	
Differentiation			
Well/moderate	34	12	0.015
Poor	18	30	
Lymphatic metastasis			
Yes	13	31	0.008
No	39	11	
Distant metastasis			
Yes	23	32	0.132
No	29	10	

A chi-square test was used for comparing groups between low and high circMET expression. *, p<0.05 was considered significant.

### circMET promotes the proliferation and metastasis of NSCLC cells

**Figure 2:**
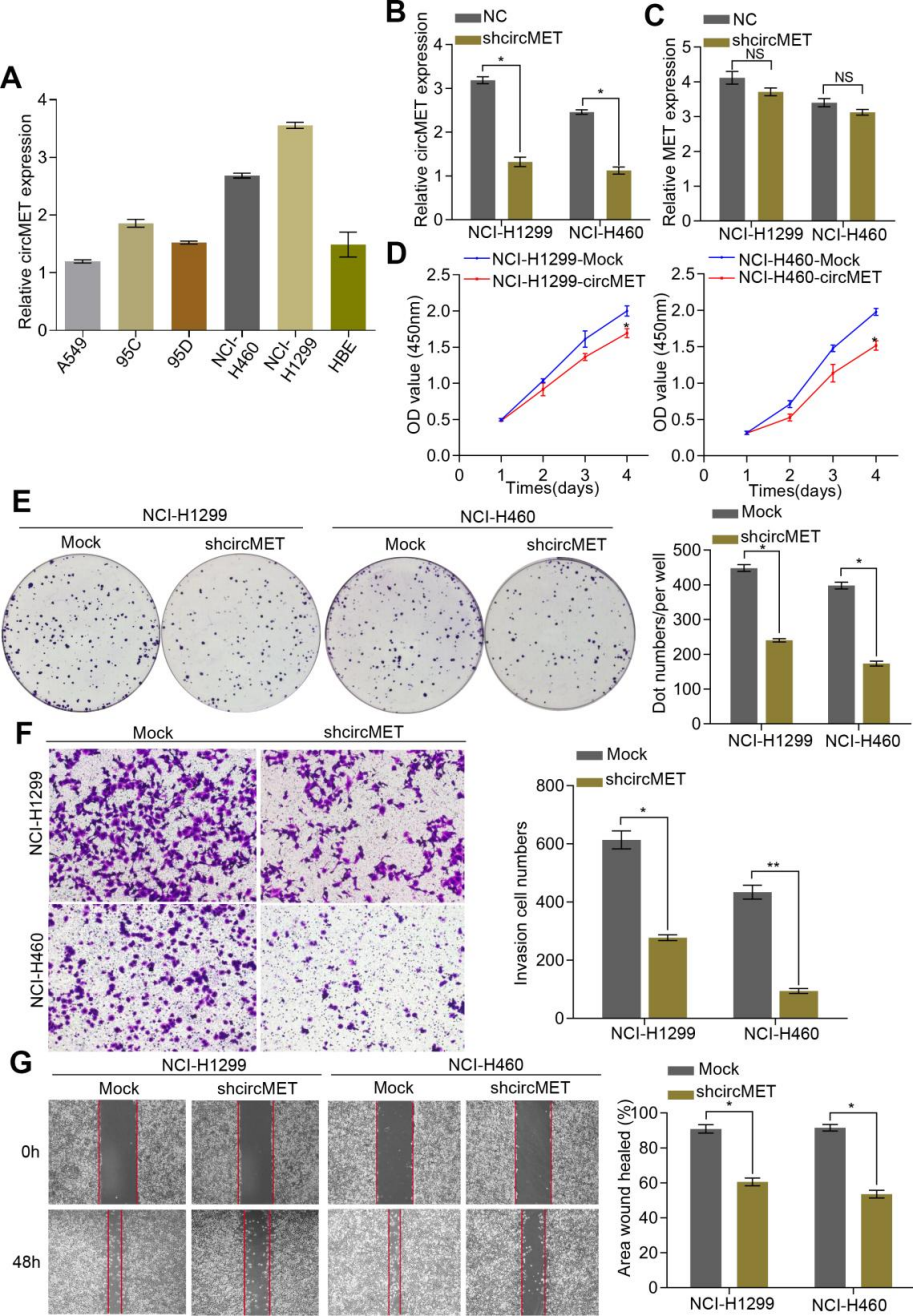
**circMET exerts oncogenic effects in NSCLC cell lines.** (**A**) Relative expression of circMET in several human NSCLC cell lines was examined by qRT-PCR normalized to GAPDH expression. (**B**) and (**C**) The efficiency of transfection in the NCI-H1299 and NCI-H460 cell lines was confirmed by qRT-PCR analysis, whereas the circMET expression was unchanged. GAPDH was used as a control for loading. (**D**) and (**E**) Cell proliferation in NSCLC cells with different circMET expression was assessed by CCK-8 (**D**) and colony formation assays (**E**). (**F**) and (**G**) Cell invasion and migration abilities were assessed by Matrigel transwell (**F**) and wound healing assays (**G**), respectively. Scale bar: 100 μm. The data was represented as the mean ± SD, * p < 0.05, ** p < 0.01, *** p < 0.001. NS: no significant.

Initially, we examined circMET expression levels in a variety of human NSCLC cell lines by RT-qPCR analyses. (Figure 2A). To further characterize the biological function of circMET in NSCLC cells, we next effectively silenced circMET expression in NCI-H460 and NCI-H1299 cells by transfection with specific shRNA (sh-circMET) (Figure 2B) and successfully forced circMET expression in A549 and 95D cells using the plasmid vector (Supplementary Figure 1A). However, qRT-PCR showed that other MET splicing products, such as the MET mRNA, had no significant effects on the expression level. In vitro Cell Counting Kit-8 (CCK-8), wound healing, migration, clone formation, and invasion assays revealed that expression was significantly suppressed in circMET knockdown NCI-H460 and NCI-H1299 cell lines compared with that in the mock control cell lines (Figure 2C–2G). Conversely, overexpressing circMET promoted the growth and migration of NSCLC cells (Supplementary Figure 1B–1E).

### circMET directly sponges miR-145-5p in NSCLC cells

**Figure 3:**
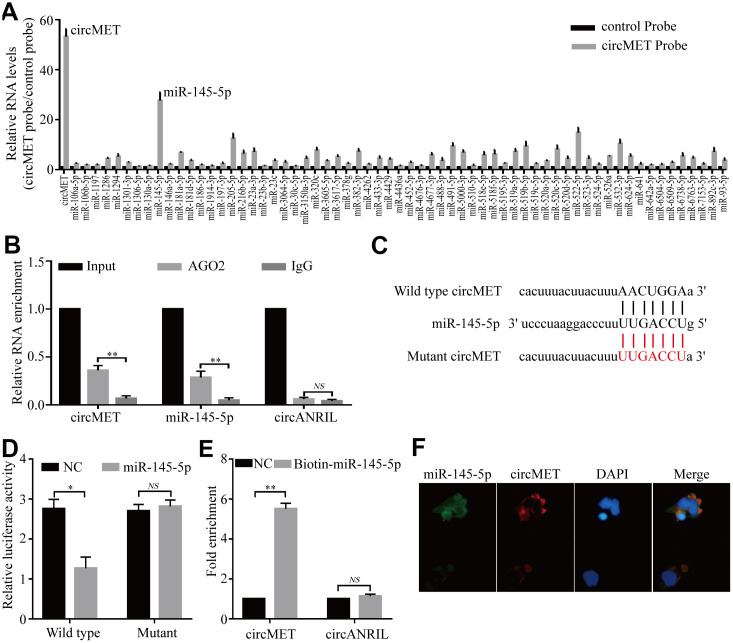
**circMET may function as a sponge for miR-145-5p.** (**A**) CircRIP experiments were performed in A549 cells using a circMET probe and NC probe. (**B**) RIP assays were performed on extracts from NSCLC cells using an antibody against AGO2. (**C**) The putative miR-145-5p binding sites with respect to circMET were predicated by StarBase v3.0. (**D**) The luciferase activity of pLG3-circMET in HEK-293T cells after cotransfection with miR-145-5p. (**E**) The level of circMET in the streptavidin-captured fractions from the NSCLC cell lysates after transfection with biotinylated miR-145-5p or control RNA (NC). cANRIL was used as a NC. (**F**) Colocalization between miR-145-5p and circMET was observed by the double FISH assay. Nuclei were stained with DAPI. Scale bar, 20um. The data was represented as the mean ± SD, * p < 0.05, ** p < 0.01, *** p < 0.001. NS: no significant.

Numerous previous studies have demonstrated that circRNAs are involved in transcriptional regulation by serving as miRNA sponges in cancer cells [24, 25]. Therefore, we attempted to explore whether circMET could bind to certain miRNAs in NSCLC progression. To confirm this idea, we next identified several potential miRNA candidates as a binding platform of circMET based on the StarBase v3.0 target prediction tool. After performing in vivo RIP with a probe specifically against circMET in A549 cells, we purified the cMET-associated RNAs by circRIP and analyzed the 63 candidate miRNAs in the complex. Interestingly, our results showed that the circMET and miR-145-5p levels were significantly enriched with the specific probe compared with those with the NC probe, while the other miRNAs had no or marginal enrichment (Figure 3A). Based on this, miR-145-5p may function as a critical miRNA that could bind to circMET in NSCLC cells.

Subsequently, RIP assays were also performed in A549 cells with antibodies against argonaute 2 (AGO2), and the results showed that circMET and miR-145-5p, but not circANRIL (a circular RNA reported not to bind to AGO2) [26, 27], were obviously enriched (Figure 3B). Thus, this result roughly suggested that circMET may provide a binding site for AGO2 and miRNAs. Next, to further elucidate the underlying mechanism, we also determined the luciferase activity of HEK-293T cells cotransfected with miR-145-5p mimics and a luciferase reporter containing wild-type or mutant target site for miR-145-5p sequences in circMET (Figure 3C). We observed that the luciferase reporter activity from only miR-145-5p with the wild-type circMET sequence had a remarkable reduction, whereas the mutant (not wild-type) luciferase reporter activity did not significantly change from that of the NC RNA (Figure 3D). Additionally, miRNA pull-down assays were performed to further explore the binding affinity between circMET and miR-145-5p by transfecting NCI-H1299 cells with biotinylated miR-145-5p mimics. The result revealed that compared with the NC, circMET was obviously enriched while circANRIL had no enrichment (Figure 3E). By performing the double FISH assay, the colocalization of circMET with miR-145-5p was visible in the merged image (Figure 3F). Overall, the above experiments illustrated that circMET could directly sponge to miR-145-5p in NSCLC cells.

### MiR-145-5p targets CXCL3 and inhibits the progression of NSCLC cells

**Figure 4:**
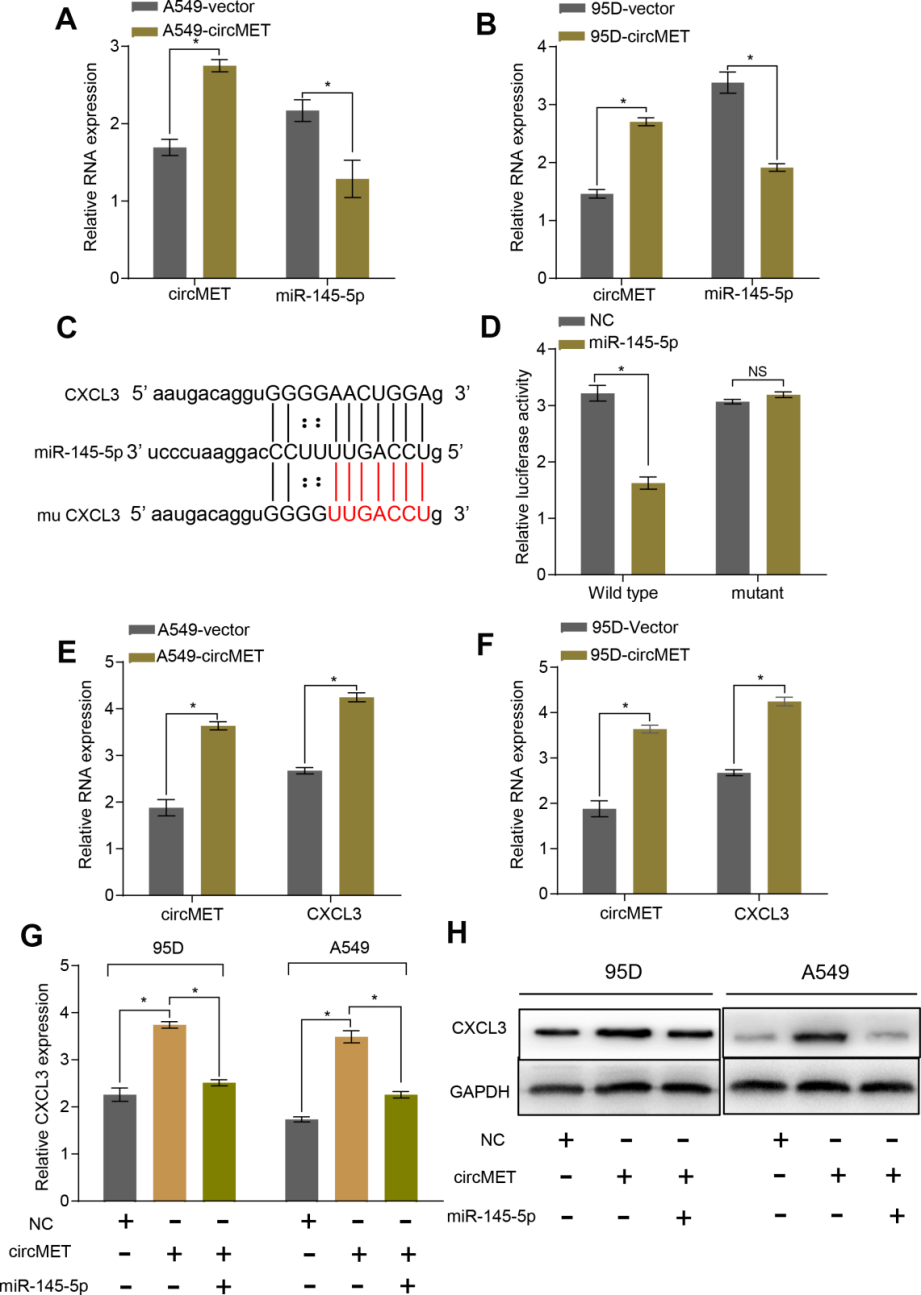
**circMET regulates miR-145-5p and targets the CXCL3 axis.** (**A**) and (**B**) RT-qPCR analyses the mRNA levels of miR-145-5p expression after overexpressing circMET in NSCLC A549 and 95D cells. GAPDH was used as a loading control. (**C**) The putative miR-145-5p binding site in the 3’UTR of CXCL3 was predicated by StarBase v3.0. (**D**) the luciferase activity of pLG3-CXCL3 in HEK-293T cells after cotransfection with miR-145-5p. (**E**) and (**F**) RT-qPCR analyses CXCL3 expression after overexpressing circMET in NSCLC A549 and 95D cells. GAPDH was used as a control for loading. (**G**) and (**H**) CircMET significantly promoted the mRNA and protein levels of CXCL3 expression, whereas the effect was retarded after upregulation of miR-145-5p. The data are represented as the mean ± SD, * p < 0.05, ** p < 0.01, *** p < 0.001. NS: no significant.

To further explore the relationship between miR-145-5p and circMET, we upregulated circMET expression in A549 and 95D cells and simultaneously measured the relative expression of miR-145-5p. However, we observed that compared with the NC cells, cells with upregulated circMET expression had strikingly decreased miR-145-5p expression (Figure 4A and 4B). In addition, miR-145-5p has been confirmed to act as a tumor suppressor in NSCLC progression (Supplementary Data1, Data 2A-2C and Data 3).. Given that miRNAs have been reported to play a pivotal part in posttranscriptional regulation by directly targeting the 3-untranslated region (3’ UTR) of target mRNAs, we tried to predict the target mRNAs of miR-145-5p through several bioinformatics analyses, including StarBase v3.0, PITA, and miRanda algorithms [28]. Ultimately, the predicted 3’ UTR of CXCL3 mRNA was confirmed to contain target sequences corresponding to miR-145-5p (Figure 4C). Next, we performed a luciferase reporter gene assay to verify whether the 3’ UTR of CXCL3 mRNA was a binding site for miR-145-5p in NSCLC cells. We constructed pLG3 luciferase reporter plasmids individually containing wild-type (wt) or mutant (mu) 3’ UTR sequences of CXCL3. The luciferase activity was significantly reduced after cotransfection with miR-145-5p mimics and the pLG3 luciferase reporter containing the wt 3’ UTR sequence of CXCL3 into HEK-293T cells. Conversely, the luciferase activity was not inhibited by the miR-145-5p mimics in mu 3’UTR sequence-transfected HEK-293T cells (Figure 4D). Therefore, these observations demonstrate that miR-145-5p could inhibit the progression of NSCLC cells, at least partly, by directly binding to the 3-untranslated region (3’ UTR) of CXCL3 mRNAs.

### circMET promotes the progression and immune evasion of NSCLC by sponging miR-145-5p to regulate CXCL3

**Figure 5:**
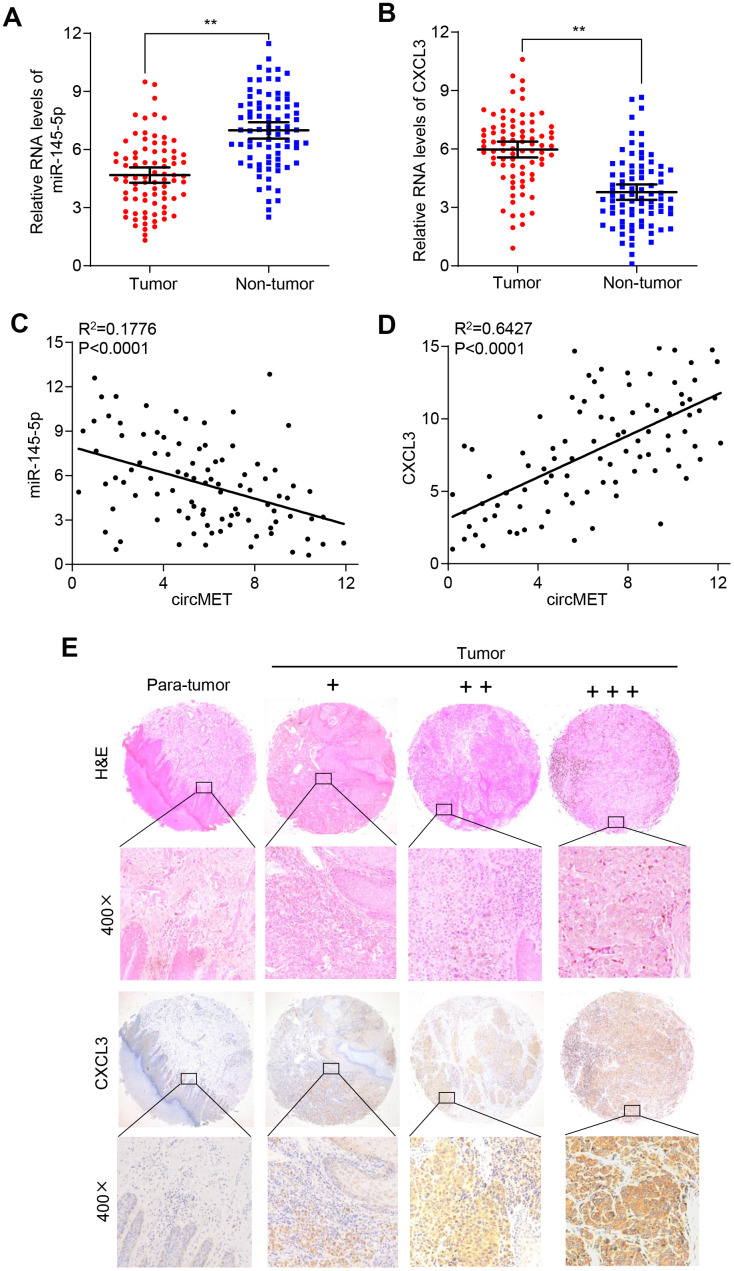
**CXCL3 exerts oncogenic effects in NSCLC tissues.** (**A**) and (**B**) miR-145-5p and CXCL3 expression were detected in 94 paired NSCLC tissues and adjacent normal tissues using qRT-PCR. GAPDH was used as a control for loading. (**C**) A negative correlation between miR-145-5p and circMET was observed in NSCLC tissues. (**D**) A positive correlation between circMET and CXCL3 was observed in NSCLC tissues. (**E**) Representative images of TMA stained with H&E and IHC for CXCL3 expression in NSCLC tissues and adjacent nontumor tissues (−, absent; +, weak; ++, moderate; and +++, strong). Scale bar: 100um. The data are represented as the mean ± SD, * p < 0.05, ** p < 0.01, *** p < 0.001.

To verify whether circMET expression could affect the levels of CXCL3 mRNA and protein in NSCLC progression, qRT-PCR and Western blot analyses were individually performed to examine their changes after overexpressing or silencing circMET and miR-145-5p in NSCLC cells. As expected, we found that the mRNA and protein levels of CXCL3 significantly increased after knockdown of miR-145-5p expression in A549 and 95D cells. Consistent with this, the circMET and CXCL3 expression levels were significantly increased after transfection of the circMET overexpression vector into NSCLC A549 and 95D cells (Figure 4E and 4F). Interestingly, the resulting increase in the CXCL3 mRNA and protein levels induced by circMET overexpression could be partially reversed by upregulation of miR-145-5p (Figure 4G and 4H). Next, using qRT-PCR analysis, we also investigated miR-145-5p and CXCL3 expression in 94 paired NSCLC tissues and their corresponding adjacent normal tissues. The results indicated that miR-145-5p expression was significantly downregulated in NSCLC tissues (Figure 5A). Conversely, the expression of CXCL3 was elevated (Figure 5B). Furthermore, negative correlations between circMET and miR-145-5p were observed in the NSCLC tissues (Figure 5C). In contrast, correlations between circMET and CXCL3 had the opposite trend (Figure 5D). Then, we focused on CXCL3 protein expression between NSCLC tissues and paired adjacent normal tissues using TMAs. Not surprisingly, the overall protein level of CXCL3 in NSCLC tissues was markedly higher than that in matched adjacent normal tissues by H&E and immunohistochemical staining. Notably, the heterogeneity of the CXCL3 levels was observed in tumor samples (−, absent; +, weak; ++, moderate; and +++, strong) (Figure 5E). Furthermore, the oncogenetic role of CXCL3 had been verified in The Cancer Genome Atlas (TCGA) dataset (Supplementary Data 2D-2G and Data 4). Collectively, all of the above experiments indicated that circMET promoted the growth and metastasis of NSCLC via the miR-145-5p/CXCL3 pathway.

To further examine the associations between the circMET/CXCL3 axis and immune escape, we analyzed the infiltration of CD8+ T cells in 94 paired of NSCLC tissues and matched nontumor tissues. Interestingly, the amounts of CD8+ T cells was significantly lower in the NSCLC tissues (Supplementary Figure 2A). The scatter plot analysis showed that negative correlations between circMET or CXCL3 expression and CD8+ T cell were frequency observed in the NSCLC tissues (Supplementary Figure 2B and 2D). On the contrary, the results revealed a positive correlation between miR-145-3p expression and CD8+ T cell in the NSCLC tissues (Supplementary Figure 2C).

### CXCL3 knockdown inhibited circMET-induced cell proliferation and migration of NSCLC cells

**Figure 6:**
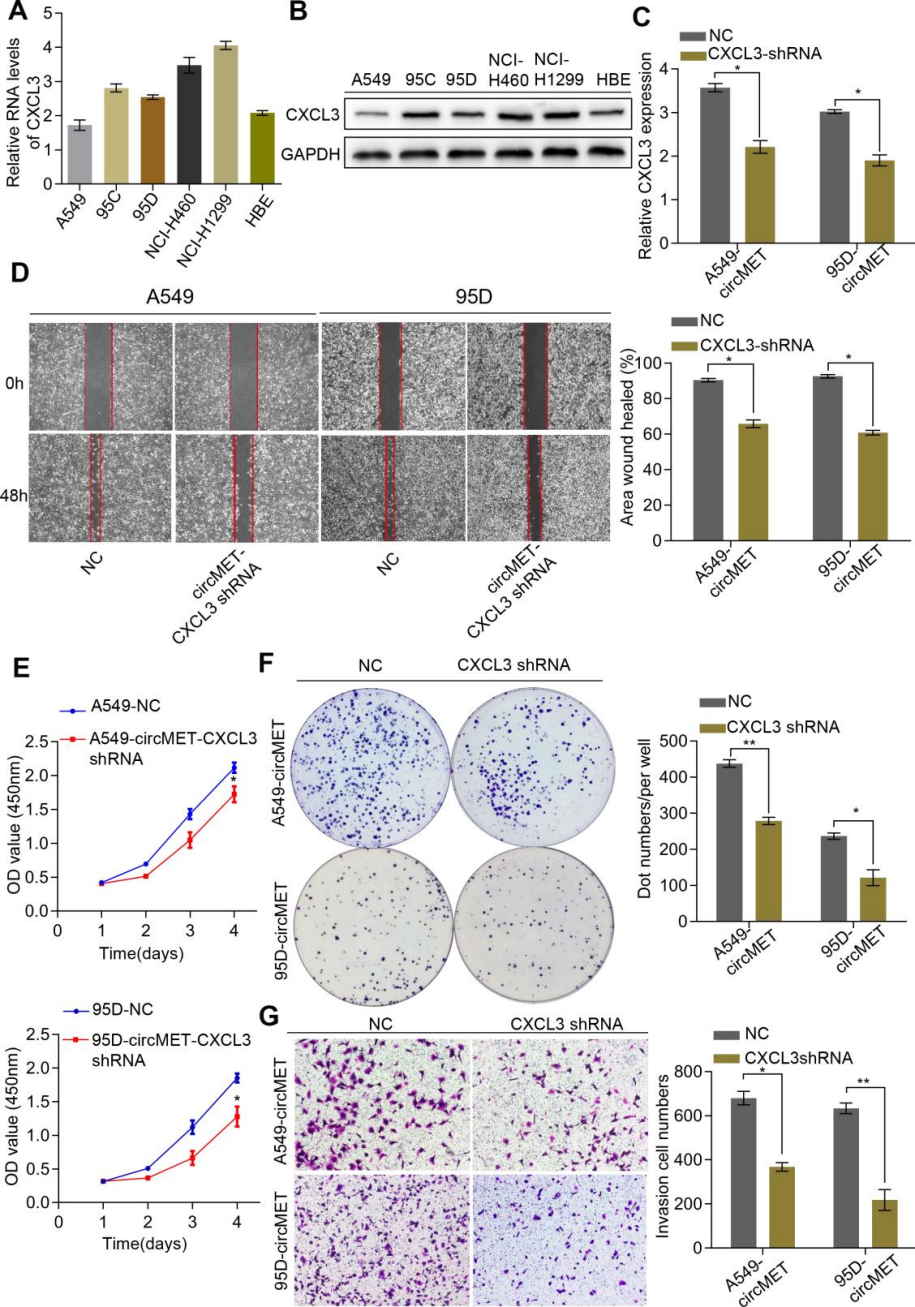
**CXCL3 knockdown inhibited circMET-induced NSCLC progression in vitro.** (**A**) and (**B**) Relative mRNA and protein expression of CXCL3 in several human NSCLC cell lines were examined by qRT-PCR (**A**) and Western blots (**B**). (**C**) The efficiency of transfection in the A549-circMET and 95D-circMET cell lines with CXCL3 shRNA plasmid were individually confirmed using qRT-PCR. GAPDH was used as a loading control. (**D**) Representative images of wound healing assay in CXCL3 knockout NSCLC cells with or without circMET overexpression. (**E**) and (**F**) Representative images of CCK-8 assay (**E**) and colony formation assay (**F**) in CXCL3 knockout NSCLC cells with or without circMET overexpression. (**G**) Representative images of the Matrigel transwell assay (**G**) in CXCL3 knockout NSCLC cells with or without circMET overexpression. Scale bar: 100um. The data are represented as the mean ± SD, * p < 0.05, ** p < 0.01, *** p < 0.001.

Additionally, we also detected CXCL3 mRNA and protein levels in a variety of human NSCLC cell lines (Figure 6A and 6B). Thus, we constructed a CXCL3 shRNA plasmid and detected the CXCL3 mRNA levels after transfection into NSCLC A549-circMET and 95D-circMET cells (Figure 6C). The results of the in vitro Cell Counting Kit-8 (CCK-8), wound healing, migration, clone formation, and invasion assays showed that compared with the NC cells, the A549-circMET and 95D-circMET cells with CXCL3 knocked down had significantly decreased malignant phenotypes (Figure 6D–6G). Taken together, these data suggested that CXCL3 knockout significantly inhibited NSCLC cell proliferation and migration induced by circMET and that CXCL3 was likely to be recognized as a potential diagnostic and prognostic biomarker for NSCLC patients.

## DISCUSSION

Since demonstration of the diverse and critical roles of circRNAs in the pathogenesis of various malignancies and the fact that only a few of them have been adequately investigated, a more in-depth analysis of the underlying molecular mechanism of circRNAs and exploring novel therapeutic targets is urgent. Several previous studies have reported that circRNAs can function as sponges for miRNAs to regulate the expression of multiple downstream mRNAs [29, 30], thereby promoting proliferation and invasion in malignant tumors, including NSCLC. Herein, we identified for the first time that circRNA MET plays a vital role in NSCLC progression. In this study, we observed that circMET expression was differentially upregulated in NSCLC tissues compared to circMET expression in adjacent normal tissues. Furthermore, high levels of circMET were predictive of aggressive clinicopathological characteristics and poorer prognosis for NSCLC patients. In particular, positive correlations between circMET upregulation and larger tumor size, lymph node metastasis, unfavorable tumor differentiation, and poor survival were verified in the NSCLC samples, indicating that circMET functions as an oncogene and novel prognostic biomarker in NSCLC tumor progression.

As acknowledged, accumulated evidence has shown that dysregulation of MET signaling contributes to several oncological processes, including processes in NSCLC cells [22, 31, 32]. Unfortunately, the primary biological function of circMET has been unknown in tumors. Here, we confirmed the result that circMET participated in promoting NSCLC cell migration, proliferation, and invasion and was not simply a byproduct of splicing [24, 27]. As mentioned above, circRNAs have been known to regulate tumor progression by sponging miRNA [6, 33]. Hence, we also demonstrated that circMET possessed the strong capability of binding to miR-145-5p, a well-studied tumor suppressor in multiple cancers, including NSCLC cells. Further, this conclusion was supported again by performing dual-luciferase assays, RNA-binding protein immunoprecipitation assays and RNA pull-down assays. Subsequently, we found that CXCL3, a potential target gene of miR-145-5p, was predicted using bioinformatics analysis. At the same time, the luciferase reporter gene assay consistently showed that miR-145-5p inhibited luciferase activity in cells transfected with the wt 3’ UTR sequence of CXCL3 but had no significant change on the luciferase activity in HEK-293T cells transfected with the mu 3’ UTR sequence. Thus, these results demonstrated that miR-145-5p directly interacted with the 3-untranslated region (3’ UTR) of CXCL3 mRNAs. Moreover, we observed that the mRNA and protein expression levels of CXCK3 were upregulated in NSCLC tissues relative to those in paired adjacent normal lung tissues. CXCL3, a well-known oncogene, has been confirmed to promote pathological progression in various malignancies, such as colorectal cancer (CC), prostate cancer, breast cancer and neuroblastoma tumors [34–37]. To confirm the correlation between circMET and CXCL3, we demonstrated that compared with the mock control cells, NSCLC cells with forced circMET expression and CXCL3 knockdown had significantly inhibited circMET-induced cell proliferation, migration, and invasion. Collectively, these observations indicate that circMET primarily mediates its promotion effect on NSCLC progression by sponging miR-145-5p to regulate CXCL3 expression.

In summary, the results presented show that circMET expression is significantly upregulated in NSCLC tissues and serves as a useful prognostic biomarker in patients with NSCLC who have received curative resection. Furthermore, we have preliminarily confirmed that circMET could enhance the proliferation and metastasis of NSCLC cells through the miR-145-5p/CXCL3 axis, indicating its oncogenic role in NSCLC progression. Taken together, our study suggests that circMET might be considered a novel diagnostic biomarker and potential therapeutic target in NSCLC treatment.

## MATERIALS AND METHODS

### Patients and specimens

In total, 94 pairs of NSCLC tissues and corresponding adjacent nontumoral tissues were obtained from patients who underwent surgery at the Second Affiliated Hospital of Nanchang University. The resected specimens of NSCLC were stained with hematoxylin and eosin (H&E) and were identified by two pathologists. The detailed clinicopathological characteristics are summarized in Table 1. All patients gave written informed consent for the collection and use of their tissue samples. The study was approved by the Ethical Review Committee of the Second Affiliated Hospital of Nanchang University.

### Cell culture

Human NSCLC cell lines A549, 95C, 95D, HBE, NCI-H1299, and NCI-H460 were purchased from Cell Bank of the Chinese Academy of Sciences (Shanghai, China). These cells were routinely cultured in DMEM or RPMI-1640 medium (HyClone, USA) (HyClone, Logan City, USA) supplemented with 10% fetal bovine serum (Gibco, Carlsbad, USA) and 1% penicillin/streptomycin (100 IU/ml) at 37°C in a humidified incubator with 5% CO_2_.

### Total RNA extraction and qRT-PCR detection

Total RNA from tissues and cultured cells was extracted using TRIzol Reagent (Invitrogen, USA) and reverse transcribed into cDNA using a PrimeScript RT Reagent Kit (TaKaRa, Japan) according to the manufacturer’s instructions. Then, quantitative real-time polymerase chain reaction (qRT-PCR) was performed with SYBR Green Real-time PCR Master Mix (Yeasen, Shanghai, China) following the manufacturer’s instructions. Glyceraldehyde-3-phosphate dehydrogenase (GAPDH) was used as an internal reference gene for quantification of circRNA and mRNA. The relative RNA expression levels were analyzed by utilizing the 2^-ΔΔCt^ method. Each reaction was conducted in triplicate independently.

### Transfection experiment

According to the manufacturer’s instructions, the NSCLC cells were transfected using Lipofectamine 2000 (Invitrogen, Carlsbad, CA) in 6-well plates at 50 to 60% confluence. The siRNAs against circMET and the CXCL3 mimics utilized for transfection were from GeneCopoeia (Shanghai, China). MiR-145-5p mimics were from RiboBio (Guangzhou, China). The adenovirus-mediated increase and knockdown of circMET or other RNA expression were designed by Genomeditech (Shanghai, China). Empty vectors were used as negative controls. Three independent experiments were performed.

### Tissue microarrays and immunohistochemistry (IHC) staining

Tissue microarrays (TMAs) of 94 pairs of NSCLC tissues and corresponding peritumoral tissues were constructed by Shanghai Biochip Co., Ltd. (Shanghai, China). The immunohistochemistry (IHC) assay was performed following a standard protocol. In brief, all paraffin tissue slides of the human lung cancer tissues were dewaxed and rehydrated. After antigen retrieval, endogenous peroxidase activity was blocked with 3% H_2_O_2_ at room temperature for 30 min. To block nonspecific binding sites, 5% bovine serum albumin (BSA) (YESEN, Shanghai, China) was incubated for 1 h at room temperature. Subsequently, the sections were incubated with a primary rabbit polyclonal antibody overnight at 4°C, followed by incubation with the corresponding biotinylated secondary antibody for 1 h at room temperature. Next, the chromogen reaction was visualized by staining slides with diaminobenzidine (DAB)-H_2_O_2_ (Gene Tech, Shanghai, China) and lightly counterstained with hematoxylin according to the manufacturer’s instructions. Finally, the slides were sealed with a cover slip with neutral balsam (Yeasen, Shanghai, China) and examined under a fluorescence microscope.

### circRNA precipitation, RNA immunoprecipitation (RIP), and luciferase reporter assays

circRNA in vivo precipitation (circRIP), RIP experiments, and luciferase reporter assays were performed as described [38]. For circRIP and RIP experiments, the A549 cells were cotransfected with the corresponding plasmids and miRNA mimics using Lipofectamine 2000. After treatment, the harvested cells were directly lysed using radioimmunoprecipitation assay (RIPA) buffer (Beyotime Biotechnology Co., Ltd., Shanghai, China) with protease and RNase inhibitors. Then, the clear supernatant was incubated with the corresponding probes for coprecipitation at 4°C overnight. After treatment with proteinase K, the immunoprecipitated RNAs were extracted by a RNeasy MinElute Cleanup Kit (Qiagen) according to the manufacturer’s instructions. Additionally, the luciferase activity was examined using the dual-luciferase reporter gene analysis system (Promega Corp., Madison, WI, USA) according to the manufacturer’s instructions.

### Fluorescence in situ hybridization (FISH) assays

The double FISH assays were performed in lung cancer cells as previously described. Briefly, biotin- or digoxin-labeled RNA probes were used to detect the colocalization of circMET and miR-145-5p according to the manufacturer’s protocol. In addition, the signals of biotin-labeled probes specific to the circMET sequence and digoxygenin (DIG)-labeled miR-145-5p probes were detected using Cy5-streptavidin (Life Technologies) and tyramide-conjugated Alexa 488 fluorochrome TSA kits, respectively. Next, for nuclear counterstaining, the cells were incubated for 15 min with 4,6-diamidino-2-phenylindole (DAPI, blue). Finally, at least five randomly selected fields per cover slip were observed and photographed by a confocal fluorescence microscope (LSM510; Zeiss, Germany).

### Biotin-labeled miRNA pull-down assay

The RNA pull-down experiments were carried out by using a Pierce Magnetic RNA-Protein Pull-Down Kit (Thermo, Waltham, MA, USA) according to the manufacturer’s instructions. Briefly, biotin-coupled miR-145-5p mimics or miRNA negative control (NC) mimics (GenePharma, China) were transfected into A549 cells and harvested 48 h later. After the biotin-coupled RNA complex was pulled down, the abundances of circMET and miR-145-5p were measured by qRT-PCR analysis.

### Colony formation and Cell Counting Kit-8 (CCK-8) assay

The clone formation and CCK-8 assay were employed to evaluate cell proliferation together. For clone formation, NCI-H1299 and NCI-H460 cells were seeded into 6-well plates at 1,000 cells/well (Corning, NY, USA) and incubated for 10 days in RPMI 1640 medium containing 10% FBS. After that, the cell clusters were rinsed with phosphate-buffered saline, fixed with 4% paraformaldehyde, and stained with 0.4% crystal violet according to the manufacturer’s instructions. Next, the number of cell clusters was observed and counted. Additionally, the Cell Counting Kit-8 (CCK-8, Yeasen, Shanghai, China) was another well-known method for detecting cell proliferation and was performed according to the manufacturer’s protocol. In brief, cells were seeded into 96-well plates at 1,000 cells/well (Corning, NY, USA). Then, 10 μl of CCK-8 reagent was added to each well after 24, 48, 72 and 96 h; the plates were incubated for 2 h; and the absorbance of all plates was measured at 450 nm.

### Wound healing, migration and transwell invasion assays

The proliferative wound healing assay and transwell assay (without Matrigel coating) were performed. Transwell assays (with Matrigel coating) (BD Biosciences, Franklin Lakes, NJ) were performed to assess cell invasion. For the wound healing assay, the cells were photographed 24 and 48 h after they were scratched with a pipette tip. For the transwell assay, 3×10^5^ cells (without Matrigel) or 10×10^5^ cells (with Matrigel) in serum-free medium were seeded into the upper chamber. After incubation for 24 h at 37 °C in a humidified atmosphere of 5% CO_2_, the number of cells that migrated to the bottom wells was counted after staining with a solution containing 0.1% crystal violet and 20% methanol. Transwell assays were performed using 24-well transwell plates (8-μm pore size, Corning, NY). Ten thousand cells seeded in serum-free medium were loaded into each upper chamber in a total volume of 200μl. Then, 0.5ml of DMEM or RPMI 1640 medium containing 20% FBS was added to the lower chamber. Then, the plates were plated in a thermostatic incubator with 5% CO_2_. Forty-eight hours later, the transwell chambers were immersed in paraformaldehyde for 10 min and then stained with a 0.5% crystal violet solution for 5 min. After that, a cotton swab was used to wipe the upper cell layer from the filter, and the cells were counted under a microscope.

### Western blot analysis

Western blot analysis was performed according to our previous study [13]. Briefly, the cell lysates were centrifuged for 15 min at 12,000 g (4°C), and supernatants containing proteins were collected. Proteins were subsequently separated using a 10% SDS-PAGE gel and then transferred onto polyvinylidene fluoride (PVDF) membranes (Millipore) by using a semidry blotter. After being sealed with 5% nonfat milk for 1 h at room temperature, the membranes were incubated overnight at 4 °C with appropriate primary antibodies, followed by incubation with HRP-conjugated secondary antibodies (Cell Signaling Technology). Next, protein expression was detected by densitometry using an enhanced chemiluminescence reagent (Amersham Pharmacia Biotech, Piscataway, NJ) according to the manufacturer’s instructions. An anti-GAPDH antibody (1:1000, Affinity, USA) served as a loading control.

### Statistical analysis

The SPSS 20.0 software program (IBM SPSS, Chicago, IL, USA) was used for statistical analyses. Comparisons between two groups were conducted using Student’s t-tests. Correlation analyses between two continuous variables were determined using the Pearson correlation coefficient. Survival curves were estimated by the Kaplan-Meier method and evaluated by the log-rank test. P<0.05 was considered statistically significant.

## Supplementary Material

Supplementary Figures

Supplementary Data 1 and 2

Supplementary Data 3

Supplementary Data 4
