# Supplementary material for: circMET promotes NSCLC cell proliferation, metastasis, and immune evasion by regulating the miR-145-5p/CXCL3 axis
Source: Aging (Albany NY). 2020 Jul 2;12(13):13038–58. doi: 10.18632/aging.103392 (PMC7377868; doi:10.18632/aging.103392)
Supplement: Supplementary Figures [file aging-12-103392-s003..pdf]

SUPPLEMENTARY FIGURES

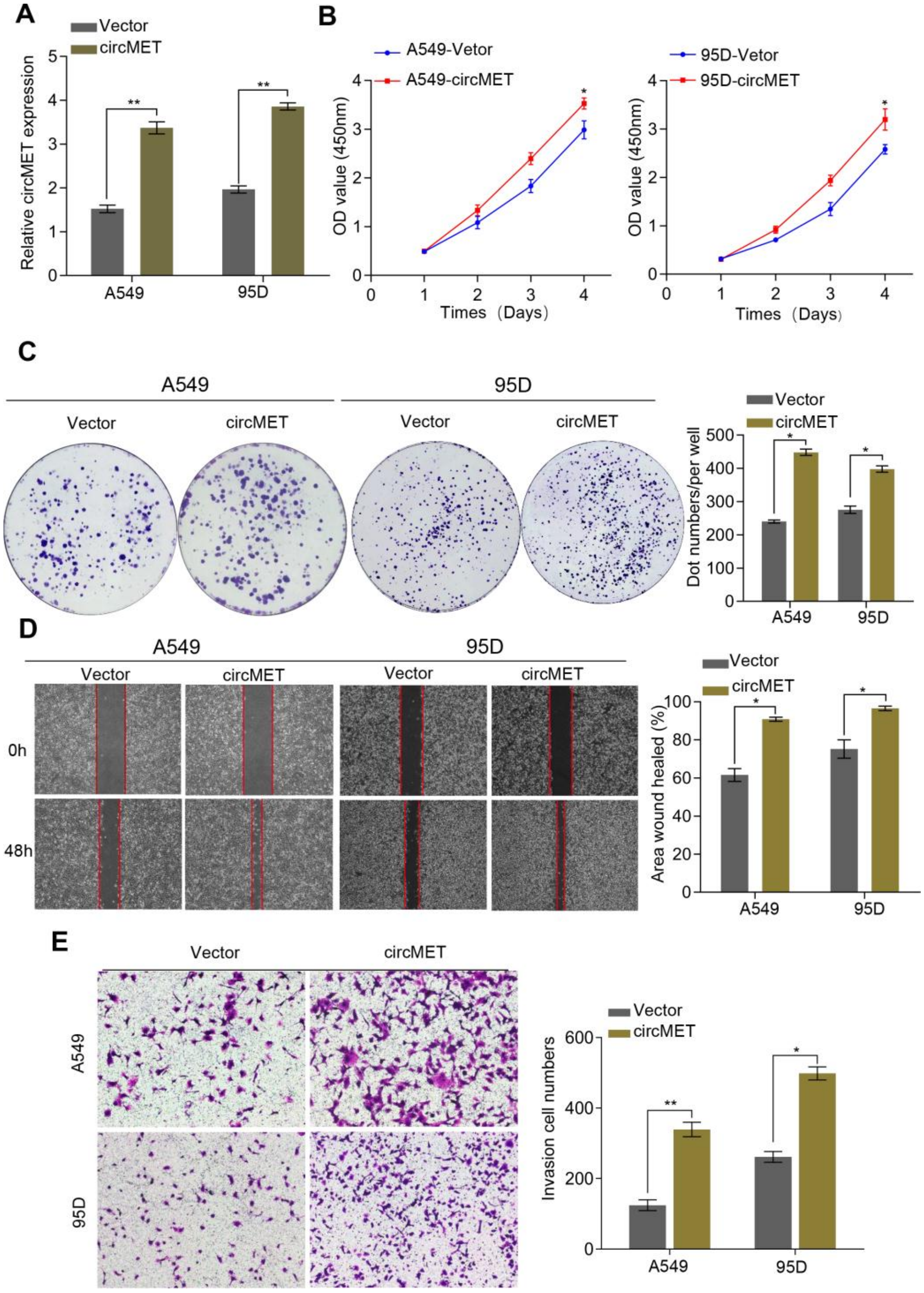

Supplementary Figure 1. circMET overexpression promotes NSCLC cell proliferation, migration, and invasion in vitro. (**A**) The circMET expression was modified in NSCLC A549 and 95D cells transfected with circMET-expressing plasmid by qRT-PCR analysis. GAPDH was used as a control for loading. (**B**) and (**C**) Representative images of CCK-8 assay (**B**) and colony formation assay (**C**) in A549 and 95D cells with or without circMET overexpression. (**D**) and (**E**) Representative images of wound healing (**D**) and Matrigel transwell assays (**E**) in A549 and 95D cells with or without circMET overexpression. The data was represented as the mean ± SD, * $p < 0.05$, ** $p < 0.01$, *** $p < 0.001$.

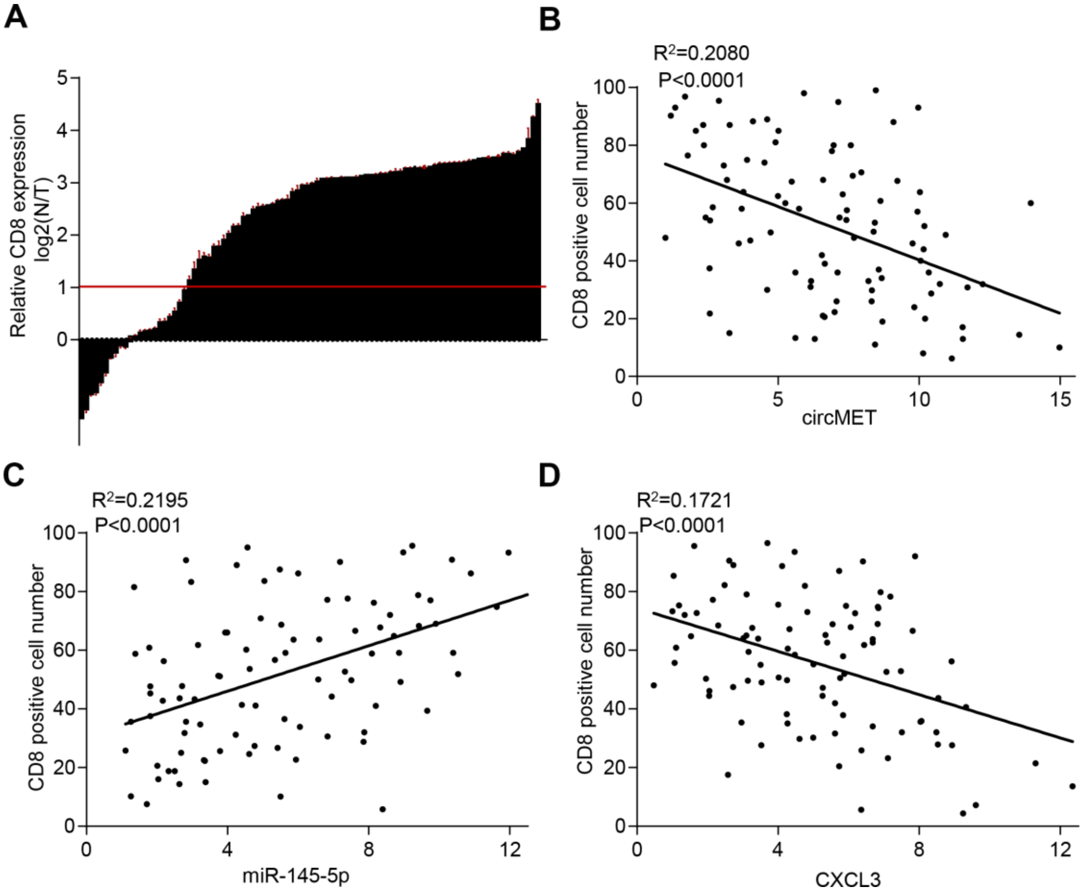

Supplementary Figure 2. circMET promotes the immune evasion in NSCLC cells. (**A**) CD8+ T cells in 94 pairs of NSCLC and matched nontumor tissues, shown as log_2(nontumor/tumor). (**B**) A negative correlation between circMET and the number of CD8-positive cells was observed in the NSCLC tissues (R^2 = 0.2080; P < 0.0001). (**C**) A positive correlation between miR-145-5p and the number of CD8-positive cells was observed in the NSCLC tissues (R^2 = 0.2195; P < 0.0001). **(D**) A negative correlation between CXCL3 and the number of CD8- positive cells was observed in the NSCLC tissues (R^2 = 0.1721; P < 0.0001).
