# Supplementary material for: circMET promotes NSCLC cell proliferation, metastasis, and immune evasion by regulating the miR-145-5p/CXCL3 axis
Source: Aging (Albany NY). 2020 Jul 2;12(13):13038–58. doi: 10.18632/aging.103392 (PMC7377868; doi:10.18632/aging.103392)
Supplement: Supplementary Data 1 and 2 [file aging-12-103392-s004..pdf]

SUPPLEMENTARY DATA

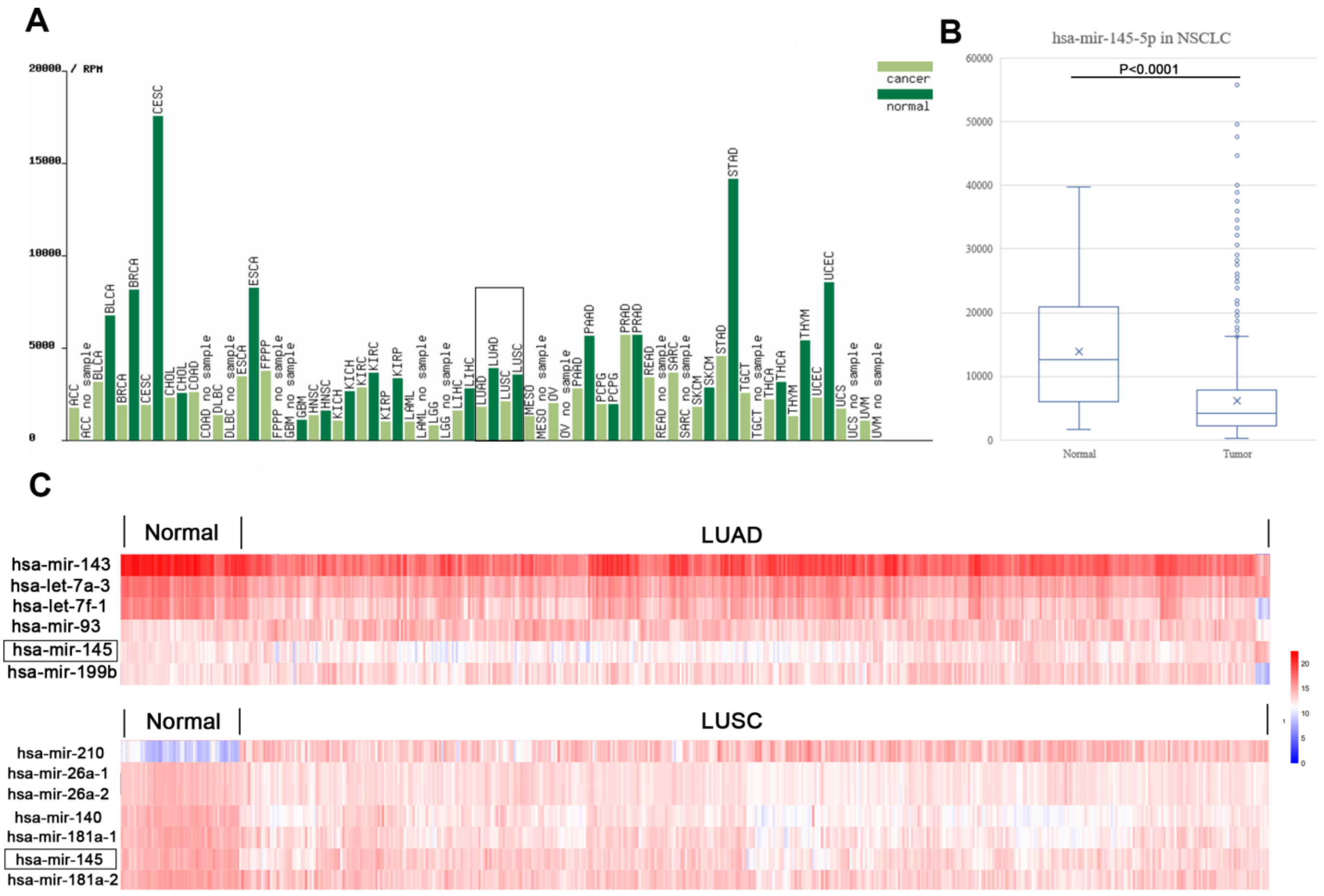

Supplementary Data 1. The expressions analysis of miR-145-5p in NSCLC from the Cancer Genome Atlas (TCGA) dataset. (**A**) The miR-145-5p expression in various types of cancer tissues and adjacent normal tissues from TCGA. (**B**) The differential expression level of miR-145-5p in NSCLC tissues and adjacent nontumor tissues. (**C**) The heatmap showed the unequal expression of miR-145-5p in LUAD compared with LUSC in TCGA.

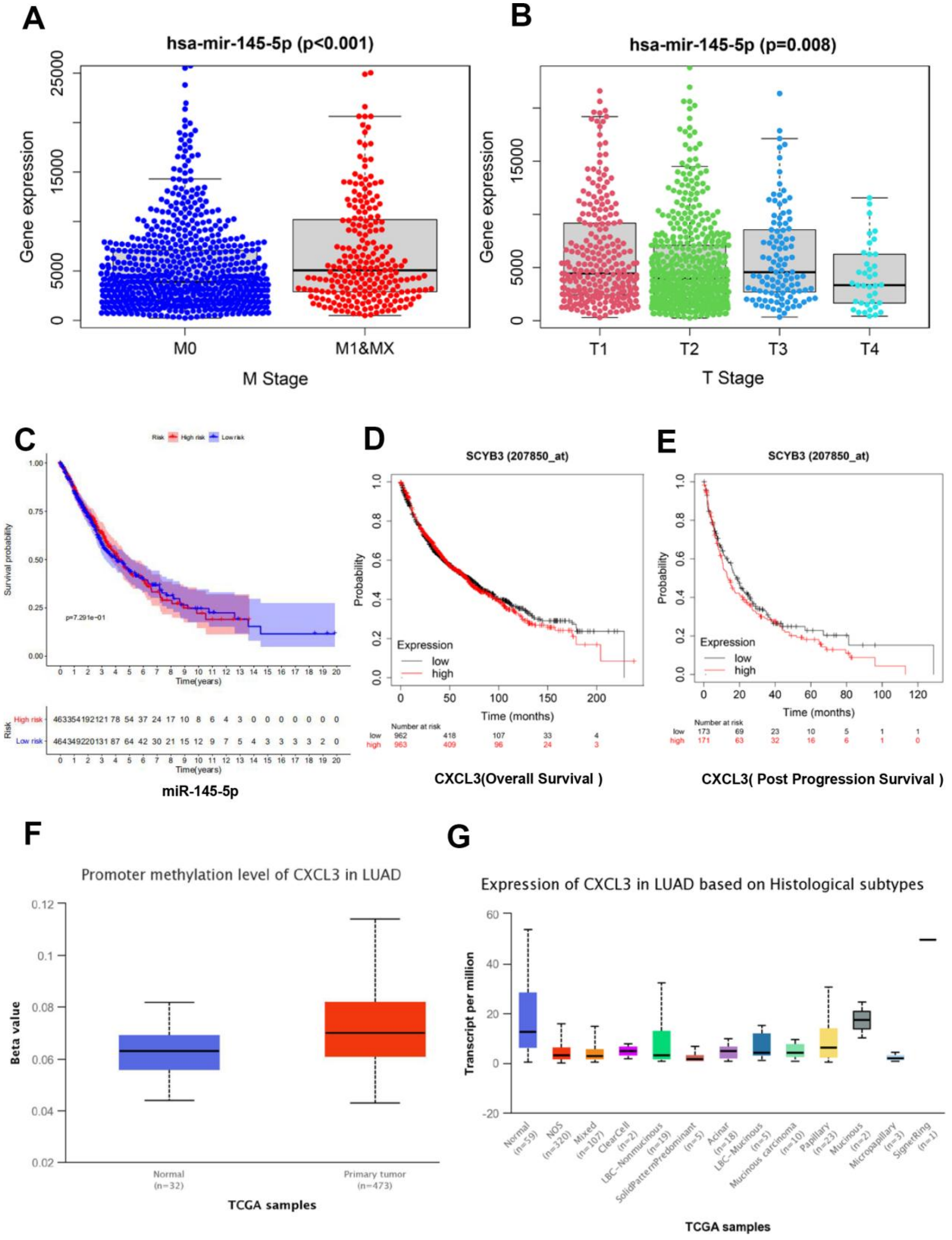

Supplementary Data 2. The expression of miR-145-5p and CXCL3 in the NSCLC tissues and prognostic significance in TCGA. (**A**) Highly hierarchical expression of miR-145-5p in the NSCLC patients with distant metastasis of tumor. (**B**) A negative correlation between malignant degree of tumor and miR-145-5p expression was observed in TCGA. (**C**) The survival probability analysis of miR-145-5p expression in TCGA. (**D**, **E**) Prognostic analysis of CXCL3 expression in TCGA. (**F**) The promoter methylation level of CXCL3 with normal and primary tumor. (**G**) The expression of CXCL3 in LUAD based on histological subtypes in TCGA.
